# Racism-critical self-reflection of professors of public health in Germany

**DOI:** 10.1093/eurpub/ckac130.153

**Published:** 2022-10-25

**Authors:** H Tezcan-Güntekin, J Simke, T Nassal, R Karls, L Löhning, T Döring

**Affiliations:** 1 Alice Salomon University of Applied Science, Department of Health and Education, Berlin, Germany; 2 Charité Berlin, Berlin School of Public Health, Berlin, Germany; 3 Universität Bielefeld, Fakultät für Soziologie, Bielefeld, Germany

## Abstract

**Background:**

In public discourse, universities are rarely understood as places of institutionalized discrimination, but rather as places of enlightenment and intercultural cosmopolitanism (Nghi Ha 2016). Existing studies focus on students’ perspectives on institutionalized discrimination. In this context, the critical self-reflection on racism is particularly relevant for people who are in positions of power, as their attitudes and actions have a direct impact on many other people, such as in the case of professors on students, academic and non-academic staff. The study reconstructs to what extent conscious or unconscious attitudes in terms of critical whiteness manifest themselves in the attitudes and, in the actions of professors in health sciences and co-constitute the realities of staff and students.

**Methods:**

Based on the critical whiteness concept according to Dietze (2009) a reconstructive, qualitative-empirical analysis (Bohnsack 2000) of eight episodic interviews (Flick 2011) with public health professors in Germany was conducted. Attitudes of professors are examined with regard to the critical reflection of their own power position in dealing with employees and students.

**Results:**

Interviewees have heterogeneous reflective skills and few structurally anchored opportunities for racism-critical self-reflection in their professional environment. The spaces are demanded by students or staff or initiated by themselves, expecting resistance from colleagues. Unconscious racism is sometimes present even with a high degree of reflexivity.

**Conclusions:**

Criticism of racism must be systematically addressed in schools of public health in order to create spaces for reflection where staff can reflect on and identify their racisms and develop collective action for racism-sensitive teaching and workplace.

**Key messages:**

- Universities need to address and work on structural racism in their institution. Structures must be the responsibility of the management level and developed participatively with staff and students.
- Spaces of reflection for university staff must be created to enable a continuous confrontation with own (often unconscious) racist actions to develop a racism-sensitive place for work/study.

